# Terpenoids from Endophytic Fungi

**DOI:** 10.3390/molecules161210604

**Published:** 2011-12-19

**Authors:** Jucimar Jorgeane de Souza, Ivo José Curcino Vieira, Edson Rodrigues-Filho, Raimundo Braz-Filho

**Affiliations:** 1 Laboratory of Chemical Science, State University of North Fluminense Darcy Ribeiro, 28013-602, Campos dos Goytacazes, RJ, Brazil; 2 Laboratory of Biochemistry of Microorganisms Micromolecules (LaBioMMi), Federal University of São Carlos, 13565-905, São Carlos, SP, Brazil; 3 Visiting Researcher — Fundação de Amparo a Pesquisa do Estado do Rio de Janeiro / State University of North Fluminense Darcy Ribeiro / Federal Rural Universty of Rio de Janeiro, Rio de Janeiro, RJ, Brazil

**Keywords:** endophytic fungus, terpenoids, antimicrobial activity

## Abstract

This work reviews the production of terpenoids by endophytic fungi and their biological activities, in period of 2006 to 2010. Sixty five sesquiterpenes, 45 diterpenes, five meroterpenes and 12 other terpenes, amounting to 127 terpenoids were isolated from endophytic fungi.

## 1. Introduction

Endophytic microorganisms are bacteria or fungi that live inside plant tissues at any moment of their life cycle, without causing damage or disease symptoms to their hosts [1,2,3,4,5]. Endophytic microorganisms can be transmitted to other plant generations by seeds or vegetative propagules, allowing wide distribution of these microorganisms in plants [6]. The colonization and propagation of these microorganisms can offer a significant benefit to their host plants by producing a plethora of metabolites that provide protection and help the survival of the plants [5]. These secondary metabolites are particularly active due to metabolic interactions with their hosts [7,8,9].

Recently, endophytes have been recognized as important sources of a variety of new biologically active secondary metabolites potentially useful for human medicine, with anticancer, antimicrobial and other activities [4,7,10,11,12], and also could be potential sources of novel natural products with industrial and agrochemical potential [5,13]. The selection of the host plant is important factor for the production of endophytic fungi and the isolation of their bioactive secondary metabolites [12,14]. This work summarizes the reported terpenoids isolated from endophytic fungi, their hosts and biological activities.

## 2. Terpenoids

### 2.1. Sesquiterpenes

**Figure 1:**
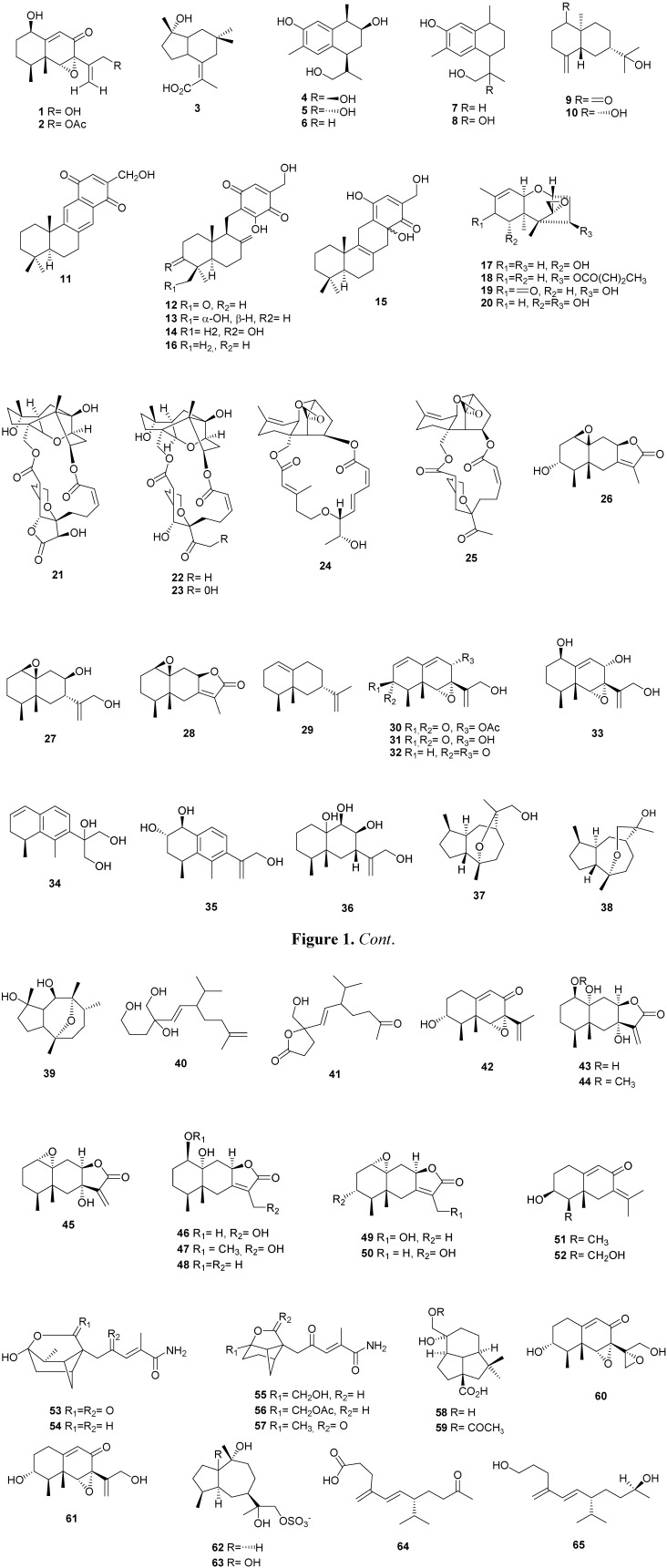
Chemical structures of sesquiterpenes from endophytic fungi.

The xylarenones A (**1**) and B (**2**), and xylarenic acid (**3**) (Figure 1) were obtained from the endophytic fungus *Xylaria* sp. NCY2, obtained from *Torreya jackii* CHUN. These compounds were tested *in vitro* in antitumor and antimicrobial assays in which they exhibited moderate antitumor activities against HeLa cells [15]. Five cadinane sesquiterpene derivatives, two diastereoisomeric 3,9,12-trihydroxycalamenenes **4** and **5**, 3,12-dihydroxycalamenene (**6**), 3,12-dihydroxycadalene (**7**) and 3,11,12-trihydroxycadalene (**8**) (Figure 1) were isolated from *Phomopsis cassia*e collected from *Cassia spectabilis* [16]. The antifungal activity of compounds **4**–**8** was evaluated against the phytopatogenic fungi *Cladosporium cladosporioids* and *C. sphaerospermum*. Compound **7** exhibited potent activity against both fungi. The cytotoxicity of the compounds **4**–**8** against HeLa cells was tested and compound **7** exhibited cytotoxicity withIC_50_ of 20 µmol/L, and **6** and **8** were weakly active (IC_50_ of 100 and 110 µmol/L, respectively). Two *ent*-eudesmane sesquiterpenes, *ent*-4(15)-eudesmen-11-ol-1-one (**9**) and *ent*-4(15)-eudesmen-1*R*, 11-diol (**10**) (Figure 1) were isolated from the endophytic fungus *Eutypella* sp. BCC 13199 from the plant *Etlingera littoralis* [17]. Five sesquiterpene quinones, (+)-(5*S*,10*S*)-4′-hydroxymethylcyclozonarone (**11**) 3-ketotauranin (**12**), 3α-hydroxytauranin (**13**), 12-hydroxytauranin (**14**) and phyllospinarone (**15**), and tauranin (**16**) (Figure 1) were isolated from *Phyllosticta spinarum*, a fungal strain endophytic in *Platycladus orientalis*. All compounds were evaluated for their cell proliferation inhibitory activity in a panel of five cancer cell lines, and only **16** showed any activity against PC-3M (prostate) and NIH 3T3 (mouse fibroblast) [18]. KLAR 5, an endophyte isolated from the twigs of *Knema laurina* (Blume) Warb., produced the trichothecene 7α-hydroxyscirpene (**17**) together with 8-deoxytrichothecin (**18**), trichothecolone (**19**) and 7α-hydroxy-trichodermol (**20**) (Figure 1). The compounds **18** and **20** were selectively active against human breast cancer (BC-1) and human small cell lung cancer (NCI-H187). Compounds **17** and **19** were moderately active against human epidermoid carcinoma of the mouth, BC-1 and NCI-H187 cancer cell lines [19]. From the *Myrothecium roridum* IFB-E009, was isolated the 10,13-cyclotrichothecane-derived macrolide myrothecine C (**21**) and *M. roridum* IFB-E012 produced two myrothecines, A (**22**) and B (**23**), mytoxin B (**24**) and roridin E (**25**) (Figure 1). These two fungi were isolated from the traditional Chinese medicinal plants, *Trachelospermum jasminoides* and *Artemisia annua*, respectively. Compounds **21**–**25** showed significant activities against the human tumor cell line nasopharyngeal epidermoid KB [20]. Four eremophilane sesquiterpenes, cupressolides A (**26**) and B (**27**), mairetolide A (**28**) and valencene (**29**) (Figure 1) has obtained of *Xylaria* sp., isolated from health tissues of *Cupressus lusitanica* [21]. *Microdiplodia* sp. KS 75-1 from the stem of *Pinus* sp. produced four eremophilane sesquiterpenes 8α-acetoxyphomadecalin C (**30**), phomadecalins C–E (**31**–**33**) (Figure 1).

Compounds **30** and **31** showed moderate activities against *Pseudomonas aeruginosa* ATCC 15442, with inhibition zones of 12 mm and 11 mm in diameter at a concentration of 200 µg/disk, respectively [22]. *Tubercularia* sp. TF5, an endophytic fungal strain isolated from the medicinal plant *Taxus mairei*, was submitted to protoplast mutations and genome shuffling, with UV and NTG, and two important mutants, M-741 and G-444, were selected for metabolites isolation and determination due to their phenotypes [23]. Three eremophilane-type sesquiterpenoids, tuberculariols A-C (**34**–**36**) (Figure 1) isolated from strain M-741, showed no inhibitory activities against *Saccharomuyces cerivisiae* ATCC9763, *Escherichia coli* CMCC44103, and *Candida albicans* AS2.538, and only weak inhibitory activity against HeLa cell line [23]. From the transformed strain G-444, five sesquiterpenoids, namely 10,11-epoxy-12-guaianol (**37**), 10,12-epoxy-11-guaianol (**38**), a backbone rearranged sesquiterpene derived from guaiane (**39**), two 1,5/1,10-diseco-guaianes (**40/41**) and sporogen-AO1 (**42**) (Figure 1) were isolated [24]. Scale up fermentation and chemical studies of *Xylaria* sp. BCC 21097, isolated from the palm *Licuala spinosa*, resulted in the isolation of eight eremophilane-type sesquiterpenes, named 1β,7α,10α-trihydroxyeremophil-11(13)-en-12,8β-olide (**43**), 7α,10α-dihydroxy-1β-methoxy-eremophil-11(13)-en-12,8β-olide (**44**), 1α,10α-epoxy-7α-hydroxyeremophil-11(13)-en-12,8β-olide (**45**), 1β,10α,13-trihydroxyeremophil-7(11)-en-12,8β-olide (**46**), 10α,13-dihydroxy-1β-methoxyeremophil-7(11)-en-12,8β-olide (**47**), mairetolide F (**48**), 1α,10α-epoxy-13-hydroxyeremophil-7(11)-en-12,8β-olide (**49**) and 1α,10α-epoxy-3r-hydroxyeremophil-7(11)-en-12,8β-olide (**50**) (Figure 1) [25]. Eremophilanolides **43**–**45**, **48**, and **49** were subjected to biological assay protocols, including cytotoxicity against cancer cell lines (KB, MCF-7, and NCI-H187), and nonmalignant Vero cells, and antimalarial (*Plasmodium falciparum* K1), antituberculosis (*Mycobacterium tuberculosis* H37Ra), and antifungal (*Candida albicans*) activities. Eremophilanolides **43**–**45** exhibited moderate cytotoxic activities in the range of IC_50_ 3.8-21 μM. Compounds **44** and **45** also displayed antimalarial activity with respective IC_50_ values of 8.1 and 13 μM. Only **45** was active against *C. albicans* (IC_50_ 7.8 μM) [25]. Isopetasol (**51**) and 15-hydroxy-3-epiisopetasol (**52**) (Figure 1) were obtained from the fungus strain CBS 121944 isolated from *Picea rubens* [26]. Tricyclic sesquiterpenoids brasilamides A-D (**53**–**56**) and pinthunamide (**57**) (Figure 1) were found in the strain of *Paraconiothyrium brasiliense* Verkley (M3-3341), collected from branches of *Acer truncatum* Bunge [27]. Compounds **54**–**56** displayed inhibitory effects on HIV-1 replication in C8166 cells, showing EC_50_ values of 108.8, 57.4 and 48.3 µM, respectively [27]. *Xylaria* sp., an endophytic fungus associated with *Piper aduncum*, produced two presilphiperfolane sesquiterpenes, 9,15-dihydroxypresilphiperfolan-4-oic acid (**58**) and 15-acetoxy-9-hydroxypresilphiperfolan-4-oic acid (**59**), along with two eremophilane sesquiterpenes, phaseolinone (**60**) and phomenone (**61**) (Figure 1) [28]. Compound (**60**) showed 20 and 50%, of cytotoxicity in CHO (Chinese hamster ovary) cell line at 20 µM and 200 µM, respectively, when compared to the DMSO treated cells. Compound **61** exhibited antifungal activity, against fungal strains *Cladosporium cladosporioides* (Fresen) de Vries SPC 140 and *C. sphaerospermum* (Perzig) SPC 491, showing a detection limit of 10.0 μg comparable with the same amount of the standard Nystatin [28]. 10,11,12-guaianetriol (**62**) and 1,10,11,12-guaianetetrol (**63**) were isolated from endophytic fungus S49 of *Cephalotaxux hainanensis* Li [29]. Two irregular sesquiterpenes, tricinonoic acid (**64**) and tricindiol (**65**) (Figure 1) isolated from *Fusarium tricinctum*, a fungus endophytic in the root tissue of the Sonoran desert plant, *Rumex hymenosepalus* [30].

### 2.2. Diterpenes

**Figure 2:**
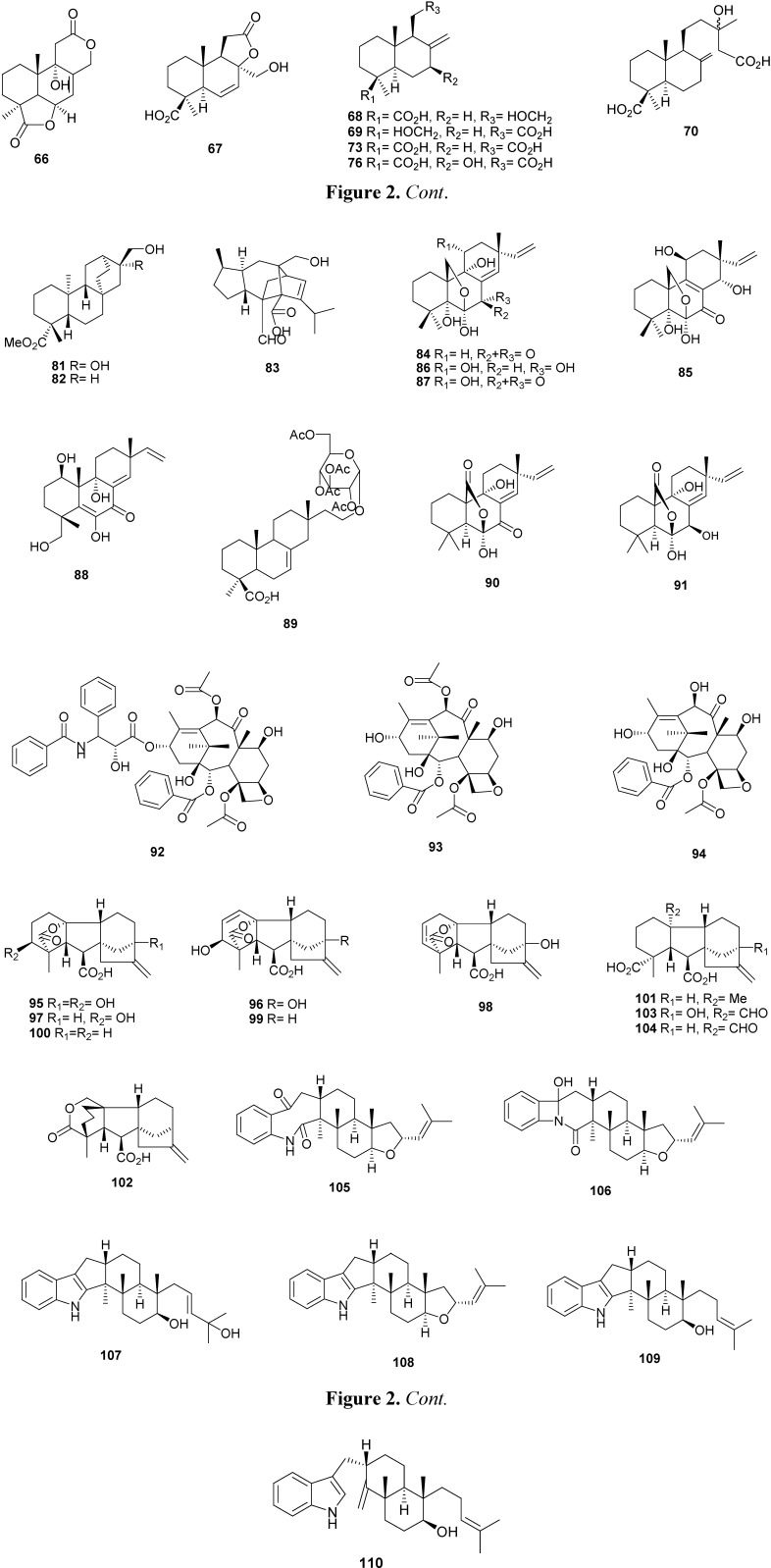
Chemical structures of diterpenes from endophytic fungi.

Botryosphaerins A–E (compounds **66**–**70**), along with 13,14,15,16-tetranorlabd-7-ene-19,6β:12,17-diolide (**71**), acrostalidic acid (**72**), acrostalic acid (**73**), agathic acid (**74**), isocupressic acid (**75**), LL-Z1271β (**76**) and CJ-14445 (**77**) (Figure 2) were isolated from the solid culture of the endophytic fungus *Botryosphaeria* sp. MHF found associated with *Maytenus hookeri* [31]. All compounds except **69** were evaluated for their inhibitory activities against several pathogenic bacterial and fungal strains. Compound **77** showed significant inhibition of *Candida albicans*, *Saccharomyces cervisiae*, and *Penicillium avellaneum* UC-4376 in comparison with nystatin which was used as a positive control [31]. Study the culture of the endophytic fungus *Fusarium* sp. WXE, isolated from *Trewia nudiflora*, resulted in the isolation of five compounds, including the three *ent*-trachylobane diterpenoids (3α)-3-hydroxy-*ent*-trachylobane-17,19-dioic acid 19-methyl ester (**78**) *ent*-trachylobane-17,19-dioic acid 19-methyl ester (**79**) and ent-trachylobane-17,19-dioic acid (**80**), and two atisane-type diterpenoids, (16α)-16,17-dihydroxy-*ent*-atisan-19-oic acid methyl ester (**81**) and 17-hydroxy-*ent*-atisan-19-oic acid methyl ester (**82**) (Figure 2) [32]. The fungi *Xylaria* sp. PSU-D14 [33] and *Eutypella scoparia* PSU-D44 [34] were isolated from the leaves of *Garcinia dulcis* and provided sordaricin (**83**) and scopararanes A (**84**) and B (**85**), and diaportheins A (**86**) and B (**87**), respectively (Figure 2). Compound **83** exhibited moderate antifungal activity against *Candida albicans* ATCC90028 with a MIC value of 32 µg/mL, and compound **87** gave the lowest MIC value of 87.8 µM against *Staphylococcus aureus* ATCC 25923. Three pimarane diterpenes, namely diaporthein B (**87**), scopararane A (**84**) and libertellenone C (**88**) (Figure 2) were isolated from the fungus *Eutypella* sp. BCC 13199 found in *Etlingera littoralis* [35]. One diterpene glycoside, 16-tetra-*O*-acetyl-D-glucopyranosyl-hymatoxin C (**89**), was isolated from the culture of the *Tubercularia* sp. TF5, an endophytic found in *Taxus mairei*, produced sphaeropsidins A (**90**) and B (**91**) (Figure 2) [24,35]. The metabolite **90** was isolated from the unidentified fungus E 99204 found in leaf of *Quercus ilex* [36]. Taxol (**92**) is a diterpenoid well known due its anticancer activities [37]. It is targeted for treat breast, lung and ovarian cancer [38,39,40]. Several endophytic fungi that produce taxol and/or taxane derivatives, baccatin III (**93**) and 10-diacetyl baccatin III (**94**) (Figure 2), have been isolated in various species of *Taxus* as *T. chinensis* [39,41,42,43,44,45], *T. cuspidate* [37,46,47,48,49], *T. media* [50], *T. mairei* [35], *T. baccata* [51] and others species of plant such as *Wrightia tinctoria* [38], *Larix leptolepis* [47], *Ginkgo biloba* [47], *Terminalia arjuna* [52], *Aegle marmelos* [53], *Cupressus* sp. [40] and *Melochia corchorifolia* L. [54]. Gibberellin (GA) is an important growth regulating metabolite, produced commercially from fungi and used in the agriculture and horticulture industry [55]. GA_1_ (**95**), GA_3_ (**96**), GA_4_ (**97**), GA_5_ (**98**), GA_7_ (**99**), GA_9_ (**100**), GA_12_ (**101**), GA_15_ (**102**), GA_19_ (**103)**, GA_24_ (**104**) (Figure 2) were identified in the endophytic *Arthrinium phaeospermum* KACC43901 [56], *Cladosporium sphaerospermum* IJL07 [57], *Aspergillus fumigates* [55], *Scolecobasidium tshawytscha* [58] isolated from the roots of *Carex kobomugi* Ohwi, *Glycine max* (L.) Merr., drought stressed cv. Hwangkeumkong and salt-stressed soybean cultivar Daewonkong, respectively. Two indoloditerpene derivatives asporyzins A (**105**) and B (**106**) and four indoloditerpenes asporyzin C (**107**), JBIR-03 (**108**), emindole SB (**109**) and emeniveol (**110**) (Figure 2) were isolated from an endophytic fungus *Aspergillus oryzae*, isolated from the marine red alga *Heterosiphonia japonica* [59]. These compounds were examined for insecticidal and antimicrobial activities and compound **108** was more active in the assay for insecticidal activity against brine shrimp (*Artemia salina*). Compound **107** exhibited potent activities against *Escherichia coli* with an inhibition diameter of 8.3 mm [59].

### 2.3. Meroterpenes

**Figure 3:**
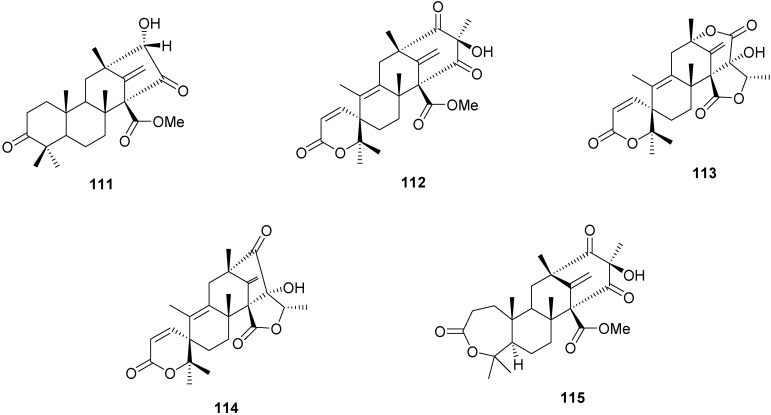
Chemical structures of meroterpenes from endophytic fungi.

The meroterpenoids preaustinoid B2 (**111**), preaustinoid A3 (**112**), austinolide (**113**) and isoaustinone (**114**) (Figure 3), were isolated from endophytic fungi *Penicillium* sp. [60] and compounds **111**, **112** and preaustinoid A1 (**115**) (Figure 3), were produced from *P. brasilianum* [61]. Both fungi were isolated from the root bark of *Melia azedarach*.

### 2.4. Other Terpenoids

**Figure 4:**
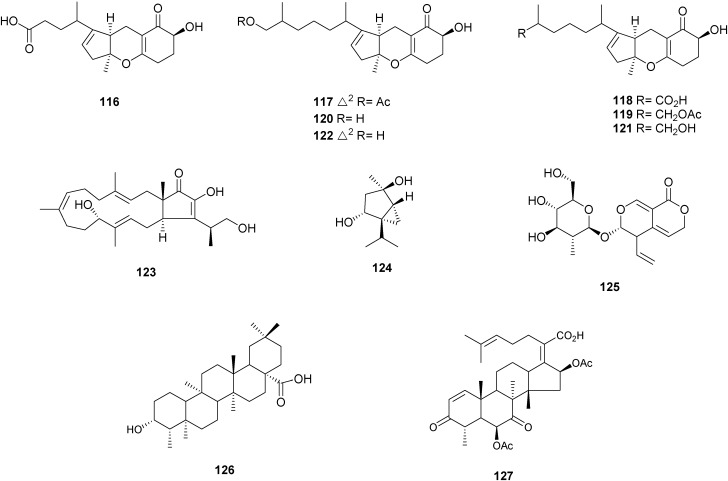
Chemical structures of meroterpenes from endophytic fungi.

Tricycloalternarenes (TCAs) A–E (compounds **116**–**120**), together with TCA 1b (**121**) and TCA 2b (**122**) (Figure 4), were isolated from the solid-state-cultured endophytic *Alternaria alternata* associated with *Maytenus hookeri* [62]. The sesterterpene terpestacin (**123**) and the monoterpene dihydroxysabinane (**124**) (Figure 4) were isolated from *Phomopsis* sp. XZ-26 collected in *Camptotheca acuminate* [9]. Two unidentified strains QJ16 and QJ18 obtained from the roots of *Gentiana macrophylla*, a traditional Chinese medicinal plant, produced the bioactive secoiridoid gentiopicrin (**125**) [63]. 3β-hydroxyfriedelan-17β-carboxylic acid (**126**) (Figure 4), a friedelan derivative, was produced by unidentified mangrove endophytic fungus No. H2K [64]. Helvolic acid (**127**) (Figure 4), a nordammarane triterpenoid isolated from *Pichia guilliermondii* Ppf9 derived from the medicinal plant *Paris polyphylla* var. *yunnanensis*, exhibited strongest antibacterial activity against all test bacteria, with MIC values ranging from 1.56 μg/mL to 50 μg/mL, and IC_50_ values from 0.98 μg/mL to 33.19 μg/mL. It also showed strong inhibitory activity on the spore germination of *Magnaporthe oryzae* with an IC_50_ value of 7.20 μg/mL [65].

## 3. Conclusions

**Figure 5:**
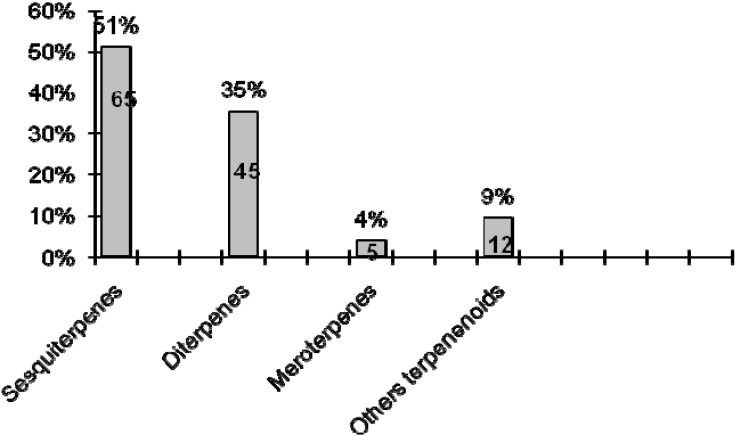
Amount and percentage of the isolated metabolites.

In Figure 5, which reports the class of terpenoids and the percentage of isolated compounds, we observe that most of the compounds isolated belong to the sesquiterpenes, which account for a total of 65 metabolites isolated.

**Figure 6:**
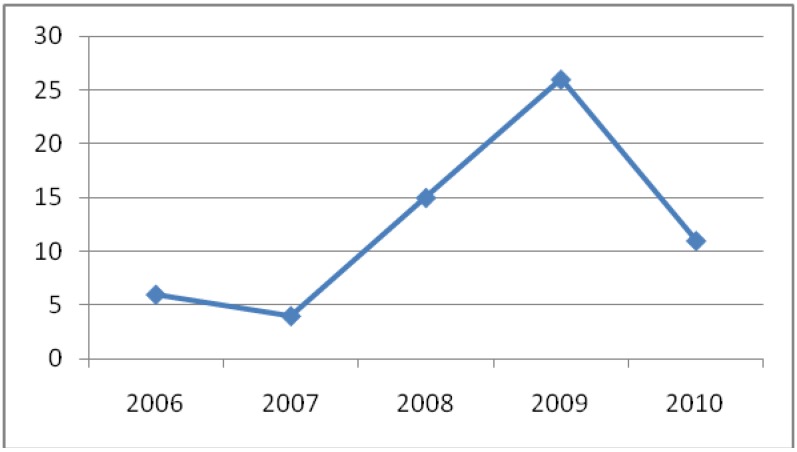
Growing of the study of endophytic fungi from 2006 to 2010.

Figure 6 shows that there was a growing interest in the number of studies on terpenoids from endophytic fungi in the period 2006 to 2010. Such studies show great promise because they indicate that work on endophytes has increased significantly in order to search for new bioactive metabolites.

Concerning the similarities observed in terpenoid structures found in endophytes and in their host plants, it appears that only a few such correlations can be identified. Among the sesquiterpenes, trichothecene macrocyclic lactones similar to those **21**–**25** were also found in *Bacharis* species [66], although it still remains to be clarified which of the associated organisms, *Baccharis* or *Fusarium*, was the sesquiterpene producer [67]. Most of the sesquiterpene found in endophytes are eremophylanes (**27**–**36**; **42**–**52**; **60**–**61**), and these appear very frequently in Xylariaceous fungi. Pimarane diterpenes with carbon skeletons similar to those of the fungal compounds **84**–**91** are also frequently found in some plant resins, especially in some *Pinus* trees [68]. Taxane diterpenoids (compounds **92**–**94**), first discovered in the plant *Taxus brevifolia* [69], have now been widely reported as fungal compounds [35,38,39,40,41,42,43,44,45,46,47,48,49,50,51,52,53,54], although it seems that none of these incredible findings has been proved yet in an interlaboratory investigation. Gibberelic acid diterpenoid compounds (e.g., **95**–**104**) are co-produced by *Fusarium* species and some plants [70]. Compounds **111**–**115** are produced by mixed biosynthetic routes in fungi, one part arising in the sesquiterpenoid and other in poliketide pathways, letting them to be called “meroterpenes” [60]. Such compounds has never been reported in plants. On the other hand, friedelane triterpenoid compounds similar to **126** are always reported from plants [71], suggesting that **126** is probably a biotransformation product of a plant triterpenoid precursor present in the cultivation medium used to grow the fungus.
